# Differences in the fruit structure and the location and content of bioactive substances in *Viburnum opulus* and *Viburnum lantana* fruits

**DOI:** 10.1007/s00709-017-1130-z

**Published:** 2017-06-10

**Authors:** Agata Konarska, Marcin Domaciuk

**Affiliations:** 1 Department of Botany, Faculty of Horticulture and Landscape Architecture, University of Life Sciences in Lublin, Akademicka 15, 20-950 Lublin, Poland; 2 Department of Plant Anatomy and Cytology, Faculty of Biology and Biotechnology, Maria Curie-Skłodowska University, Akademicka 19, 20-033 Lublin, Poland

**Keywords:** *Viburnum* fruits, Bioactive substances, Carotenoids and poliphenols, Anatomy and ultrastrastructure, Histochemistry and fluorescence

## Abstract

Many *Viburnum* species are popular ornamental shrubs and, simultaneously, highly valued medicinal plants as a source of many bioactive compounds, including antioxidants. *Viburnum* bark, flowers, and fruits are widely used in traditional and folk medicine, and the fruits of some species are used as cooking ingredients. The knowledge of the microstructure of *Viburnum* fruits and the accumulation sites of bioactive substances in these organs is rather poor. Comparative analyses of the microstructure of ripe *Viburnum opulus* and *Viburnum lantana* drupes were carried out using light, scanning, and transmission electron microscopes. The location of various groups of metabolites in the fruits of both species was determined with the use of histochemical tests and fluorescence microscopy. Additionally, the major antioxidants, i.e. carotenoids, polyphenols, and flavonoids, were quantified and a number of morphometric traits of the drupes were presented. The *V. opulus* and *V. lantana* fruits were found to differ in some morphological traits and in many characteristics of the pericarp anatomy and ultrastructure. It was shown that the *Viburnum* fruits contained lipids and lipid compounds (carotenoids, essential oils, steroids, and saponins), polyphenols (tannins, flavonoids, and anthocyanins), pectins, and proteins. The fruits of *V. opulus* contained greater quantities of carotenoids, polyphenols, flavonoids, steroids, and pectins than the *V. lantana* drupes, whereas the latter were characterised by higher contents of essential oils, saponins, and proteins. The metabolites were located in different pericarp layers, but the greatest amounts were identified in the drupe skin.

## Introduction

*Viburnum opulus* L. and *Viburnum lantana* L., i.e. representatives of the family Adoxaceae, are common in natural habitats on the European continent and in some regions of Asia and North Africa. The shrubs are primarily used as ornamental plants, although their medicinal properties have long been known. Furthermore, in many European and Asian countries, *V. opulus* fruits are considered edible (Velioglu et al. 2006; Česonienė et al. 2008; Łuczaj 2011), although raw *V. opulus* fruits have an astringent-bitter-sour taste and are considered slightly toxic because of the occurrence of saponin glycosides and viburnine (Soylak et al. 2002; Wang et al. 2008). Due to the presence of tannins, low pH, and inadequate taste, *Viburnum* fruits are not very attractive for frugivores; acting as chemical repellents, they deter predators and pathogens (Hernandez 2001; Cazetta et al. 2008).

Typically, the fruit in the family Adoxaceae is a fleshy drupe containing from three to five stones (*Sambucus*), but sometimes the drupe mesocarp is dry (*Adoxa*) (Donoghue et al. 2003). An exception is the one-seeded achene-like fruit in *Sindoxa* (Wu et al. 1981)*.* The *Viburnum* fruit is a fleshy berry-like drupe, which has a single-seeded drupelet and endospermic seeds (Jacobs et al. 2008). The drupes, ripening in August (*V. lantana*) or September (*V. opulus*), are considered persistent fruits, as they are not shed in autumn but remain on shrubs throughout winter, often until May or longer.

Although many reports of the content and chemical composition of phytochemicals produced by fruits of different *Viburnum* species are available, there is little or no scientific evidence for the fruit micromorphology, anatomy, and ultrastructure, as well as the location of these phytochemicals in the fruit cells and tissues. To date, only some morphological traits of *Viburnum* fruits have been characterised, e.g. the number of fruits per inflorescence (Česonienė et al. 2008) or on the shoot (Özrenk et al. 2011), their size (Snow and Snow 1988), weight (Cornelissen et al*.* 1996), and some rheological traits, such as dimensions, diameter, sphericity, bulk density, fruit density, volume, terminal velocity, rupture strength, and porosity (Akbulut et al. 2008), as well as the morphology and anatomy of drupelets and seeds (Jacobs et al. 2008; Kalyoncu et al. 2013). The structure of the outer layers of the pericarp forming fruit skin largely determines the resistance of these organs to adverse external factors and exerts an impact on the intensity of transpiration, which may lead to fruit wilting (Solovchenko and Merzlyak 2003; Veraverbeke et al. 2003). In turn, the presence of various pigments in skin cells determines the attractiveness of the fruit to frugivores, which has an effect on seed dispersal (Schaefer et al. 2008; Rodríguez et al. 2013). Moreover, the morphoanatomical features of fruits and seeds are a diagnostic value that can be helpful in explanation of the ontogeny, evolution, and phylogeny of the genus *Viburnum* (Donoghue 1983; Donoghue et al. 2003, 2004; Winkworth and Donoghue 2005; Jacobs et al. 2008, 2010). In turn, the identification of structures involved in production and accumulation of secondary metabolites can be used to develop strategies for maximisation of the yields of these compounds.

The aim of the study was to compare the morphological, histochemical, and ultrastructural diversity of the species and demonstrate which pericarp parts are active in the accumulation of several groups of biologically active substances, in particular the health-promoting components. Additionally, the content of the major antioxidant compounds, i.e. carotenoids, polyphenols, and flavonoids, was quantified.

## Materials and methods

The *V. lantana* L. and *V. opulus* L. shrubs originated from the Arboretum of the UMCS Botanical Garden in Lublin, SE Poland (51° 15′ 44′ N, 22° 30′ 48′ E). In 2015, the full bloom was noted in the first days of May for *V. lantana* and at the end of May for *V. opulus*. Ripe, fully stained, firm fruits (120 DPA—day post anthesis) of both species were chosen for the investigations. Ripening fruits were counted in 20 inflorescences of each species. Fruit and seed weight (*n* = 100) were determined using an analytical balance, and fruit and seed length, width, and thickness (*n* = 20) were measured with a calliper.

Transverse sections (perpendicular to the fruit axis) were incised with razors from fragments of fresh fruits with the skin sampled from the equatorial part. Next, they were analysed in water under a light microscope (and in polarised light) Nikon Eclipse E200 (Nikon, Japan) and after application of the histochemical tests under a fluorescence microscope (FLM) Nikon 90i equipped with digital camera (Nikon Fi1) and the NIS-Elements Br 2 software. Additionally, permanent preparations were made from unpeeled fruit segments and analysed under light, scanning, and transmission electron microscopes.

### Anatomy parameters

The following anatomical features of the pericarp were measured in hand-made sections (*n* = 20): the height and width of epidermis and hypodermis cells, the thickness of the cuticle, epidermis outer walls, periclinal hypodermis walls adjacent to the epidermis, endocarp-forming sclereid walls, exocarp (comprising epidermis and hypodermis), five peripheral layers of the mesocarp located directly under the skin, and endocarp. The number of mesocarp and endocarp layers was determined as well.

### Histochemistry

The main classes of metabolites in the fresh fruit sections were investigated using the following histochemical tests: Sudan III and Sudan IV for total lipids (Pearse 1968; Brundrett et al. 1991), Nile Blue (Jensen 1962) for neutral and acidic lipids, Nadi reagent (David and Carde 1964) for essential oils, potassium dichromate (Gabe 1968) for tannins, ferric trichloride (Johansen1940; Gahan 1984) for polyphenols, IKI solution (Johansen 1940; Jensen 1962) for starch and proteins, and Ruthenium Red (Johansen 1940; Jensen 1962) for pectin.

### Fluorescence

The hand-cut cross-section through fresh fruits were also examined using fluorescence microscope equipped with a FITC filter set (excitation light 465–495 nm and a barrier filter—wavelength 515–555 nm), TRITC filter set (excitation light 525–565 nm and a barrier filter—wavelength 555–600 nm), and Cy5 filter set (excitation light 590–650 nm and a barrier filter—wavelength 663–738 nm) for autofluorescence of cuticle and carotenoids, for the presence of flavonoids by induction of fluorescence with the fluorochromes aluminium chloride (Guérin et al. 1971) and magnesium acetate (Charrière-Ladreix 1976), for the presence of lipophilic substances with the fluorochromes Neutral Red (Conn 1977; Lulai and Morgan 1992), and for the presence of terpenes containing steroids with the fluorochrome antimony trichloride (Mace et al. 1974).

For all the histochemical and fluorescence methods used, standard control procedures were carried out simultaneously following the suggestions of the respective authors.

### Scanning electron microscopy

The fruit samples were not dried for the scanning electron microscopy (SEM) analyses, as conventional fixation of material used for scanning electron microscopy observations can alter or remove lipids forming the wax coating on the fruit surface (Konarska 2013). Immediately after fruit collection from the bushes, the external parts of the mature fruits with skin (*n* = 5) in the shape of cubes (3 mm × 3 mm × 2 mm) were cut out from the equatorial area perpendicularly to the main axis of the viburnum flesh with a stainless steel cutter. Next, they were mounted carefully onto aluminium stubs with a double-sided carbon tape. After coating with a 15-nm thick layer of gold, the samples were examined under a TESCAN/VEGA LMU scanning electron microscope at an accelerating voltage of 10 kV.

### Light and transmission electron microscopy

To prepare permanent semi-thin and ultra-thin sections, small cubes of the fruits with skin with a volume of ca. 4 mm^3^ (*n* = 5) were fixed in 2.5% glutaraldehyde in 0.1 M phosphate buffer at pH 7.2 for 12 h at 4 °C temperature. Next, the sections were carefully washed three times in phosphate buffer and dehydrated in an ethanol series. For transmission electron microscopy (TEM), permanent samples were additionally fixed in 1% OsO_4_ for 1.5 h and washed three times in distilled water. Next, samples for light microscopy (LM) and TEM observations were embedded in LR white resin (LR White acrylic resin, medium grade, Sigma-Aldrich) and polymerised at 60 °C. Semi-thin (a thickness range from 0.7 μm) and ultra-thin (70 nm thickness) sections were cut with glass knives with the use of a Reichert Ultracut S ultramicrotome. For general histology, semi-thin sections were stained with a 1% aqueous methylene blue-azure II solution (O’Brien and McCully 1981). Next, they were examined using a Nikon Eclipse E200 light microscope and measured with the use of the NIS-Elements Br 2 imaging software. Ultra-thin sections for TEM were stained in a 0.5% aqueous solution of uranyl acetate in 0.5% acetic acid and lead citrate (Reynolds 1963). Observations and documentation were made using the FEI Technai G2 Spirit Bio TWIN transmission electron microscope at an accelerating voltage of 120 kV. A Megaview G2 Olympus Soft Imaging Solutions camera was used for capturing the images.

### Foam test for saponins

Saponins in the fruits of the examined *Viburnum* species were detected with the so-called foam test (Klimek 2011; Pandey and Chandel 2014). Ten millilitres of boiling water were added to 0.5 g of the powdered raw material (skin or pulp) and allowed to cool down. Next, the tube with the content was shaken vigorously for ca. 10 s. The presence of saponins was evidenced by formation of a 1- to 10-cm high foam layer, which persisted for at least 10 min and did not disappear after addition of several drops of 2 n hydrochloric acid. Froth was classified for saponin content as follows: no froth = negative, froth less than 1 cm high = weakly positive, froth 1.2–2 cm = positive, and froth greater than 2 cm high = strongly positive.

### Determination of carotenoid, polyphenol, and flavonoid contents

To determine the total content of carotenoids, samples of fresh fruit flesh with the skin (10 g) were homogenised and carotenoids were extracted with 80% acetone with addition of petroleum ether (1:1) until complete discolouration of the tissues. After removal of acetone with water, the ether extract was dried with anhydrous Na_2_SO_4_ and concentrated under reduced pressure at a temperature of 35 °C. The total content of carotenoids was determined in the extract with the colorimetric method as milligrams∙100 g^−1^ of fresh weight (Rutkowska 1981; Perucka 2004).

The total polyphenol content in the methanol fruit extracts of each species was estimated by application of Folin-Ciocalteu reagent according to the colorimetric method described by Singleton and Rossi (1965). Caffeic acid was used as a standard. The polyphenol concentration was expressed as caffeic acid equivalents in grammes·100 g^−1^ of dry extract.

The flavonoid content in the fruit extracts of each species was determined with the spectrophotometer method according to the Polish Farmacopoeia (2002). Quercetin was used as a standard. The total content of flavonoids was expressed as equivalents of quercetin in grammes·100 g^−1^ of dry extract.

### Statistical analyses

Means and standard deviations (±SD) were calculated for all the measured parameters using the Excel 7.0 software (Microsoft, Redmond, Wash). Data were analysed by one-way analysis of variance (ANOVA) and Tukey’s multiple range test for comparison of means, using the Statistica 7.0 software (StatSoft, Inc., USA). Differences between species were considered statistically significant at the level of *P* ≤ 0.05.

## Results

### Morphometric traits of fruits

**Fig. 1:**
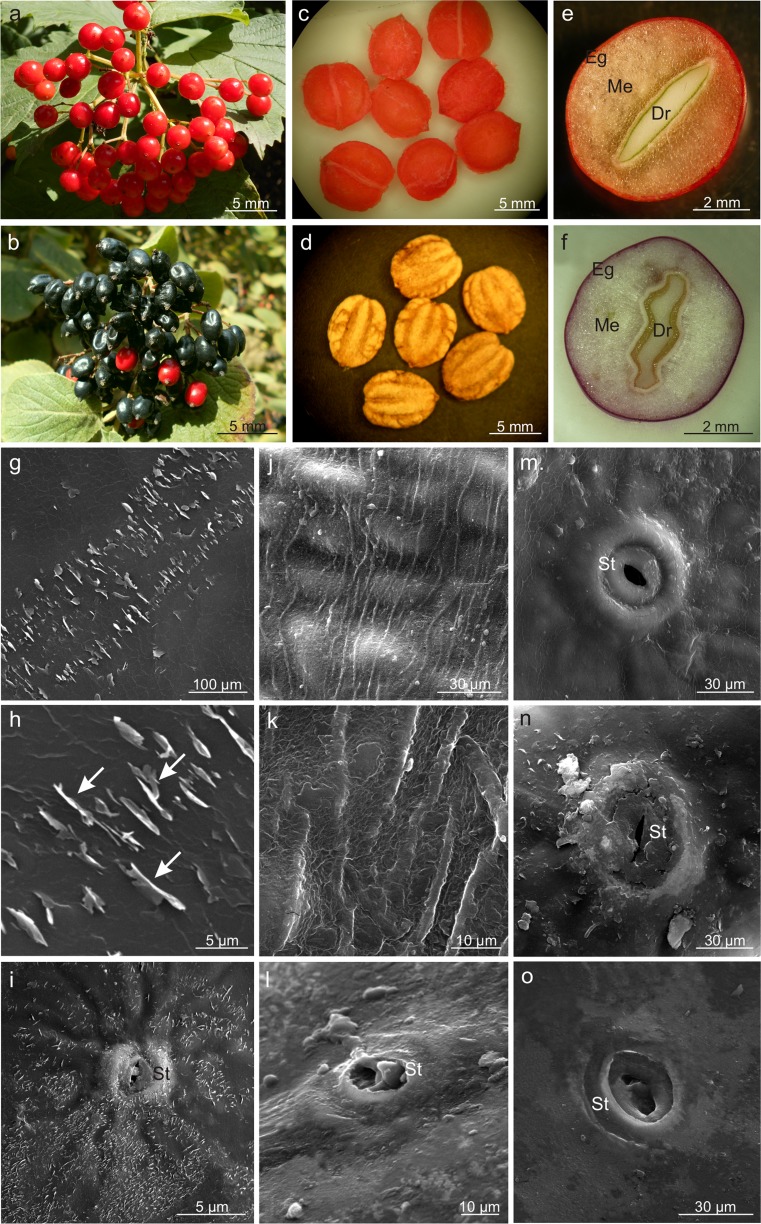
Morphology of the drupes and drupelets as well as the drupe surface of *V. opulus* (**a**, **c**, **e**, **g–i**, **m**, **n**) and *V. lantana* (**b**, **d**, **f**, **j–l**, **o**). **a**, **b** Ripened drupes collected in racemes. **c**, **d** Flattened *Viburnum* drupelets with a smooth surface (**c**) and longitudinal grooves (**d**). **e**, **f** Cross-sections through the pericarp and drupelets; note the lenticular shape (**e**) and corrugations of the drupelet (**f**). **g**–**i** Epidermis surface with numerous vertically oriented crystalline wax platelets *(arrows)*. **j**, **k** Epidermis surface with parallel cuticular striae. **i**, **m**, **n** Oval stomata located on small protuberances. **l**, **o** Lenticular stomata located at the epidermis level; *Eg* exocarp, *Me* mesocarp, *Dr* drupelet, *St* stoma

The *Viburnum* fruit is a single-seed drupe with remnants of the sepals and pistil located opposite to the fruit stalk. The ripe, almost spherical *V. opulus* fruits had an intense coral colour and were relatively firm, whereas the slightly elongated and flattened fruits of *V. lantana* were black and quickly lost firmness (Table 1; Fig. 1a, b). Compared with the *V. lantana* fruits, the number of *V. opulus* fruits in the inflorescences was by 15% lower, while the weight and size of the fruits in this species were twofold higher (Table 1). Although the seeds of both species had similar weight and size, they accounted for ca. 20% of the fruit weight in *V. opulus* and ca. 30% in *V. lantana* (Table 1). The seeds in both species were strongly flattened and were generally located slightly asymmetrically to the centre of fruit symmetry (Fig. 1c–f). The drupelet in *V. opulus* in the cross-section had a lenticular shape and the endocarp surface was smooth, whereas the cross section of the *V. lantana* drupelet exhibited corrugation and the presence of longitudinal grooves on its surface (Fig. 1c–f). The endocarp in *V. opulus* had a coral colour and consisted of five layers of drupelet cells. In *V. lantana*, it was beige-brown and had two more layers of sclereids. The thickness of the endocarps in the analysed *Viburnum* species was similar, but the lignified walls of *V. lantana* sclereids were twice as thick as in *V. opulus* (Table 1).

**Table 1:** Morphological and anatomical characteristics of *Viburnum opulus* and *V. lantana* fruits

Characteristics (*n* = 20)	*V. opulus*	*V. lantana*
Number of fruits in raceme	68.40 ± 19.86a	81.45 ± 40.58b
Weight of 100 fruits (g)	42.86 ± 3.23a	21.77 ± 1.47b
Length of fruit (mm)	11.85 ± 0.74a	10.35 ± 3.33a
Width of fruit I (mm)	9.60 ± 0.39a	9.00 ± 0.67a
Width of fruit II (mm)	9.60 ± 0.39a	8.50 ± 0.55b
Weight of 100 drupelets (g)	3.60 ± 0.24a	3.77 ± 0.52a
Length of drupelet (mm)	6.92 ± 0.57a	6.55 ± 1.42a
Width of drupelet (mm)	6.01 ± 0.22a	5.62 ± 1.24b
Thickness of drupelet (mm)	1.10 ± 0.03a	1.14 ± 0.1a
Number of the endocarp layer	5 ± 1a	7 ± 1b
Thickness of the endocarp (μm)	108.09 ± 11.94a	111.67 ± 12.87a
Thickness of the endocarp sclereid cell wall (μm)	6.13 ± 0.82	13.29 ± 2.13
Thickness of the cuticle (μm)	5.30 ± 0.2a	6.45 ± 0.34b
Thickness of the outer epidermis cell wall (μm)	3.40 ± 0.14a	2.33 ± 0.16b
Height of the epidermis cell (μm)	29.03 ± 2.91a	40.05 ± 5.90b
Width of the epidermis cell (μm)	34.84 ± 4.14a	46.60 ± 11.01b
Height of the hypodermis cell (μm)	79.93 ± 16.39a	72.70 ± 10.71b
Width of the hypodermis cell (μm)	90.90 ± 23.84a	67.24 ± 16.60b
Thickness of the hypodermis cell wall (μm)	17.76 ± 3.51a	6.7 ± 2.03b
Thickness of the skin (μm)	109.59 ± 11.62a	113.49 ± 10.26a
Number of the mesocarp parenchyma layer	5–17a	5–17a
Thickness of five mesocarp parenchyma layers (μm)	806.91 ± 101.61a	633.18 ± 108.87b

Values are mean ± SD (standard deviation). Different letters within a line mean statistically significant differences (*P* ≤ 0.05)

### Fruit micromorphology and anatomy

The surface of the drupe in *V. opulus* had a smooth cuticle with numerous horizontally and vertically oriented crystalline wax platelets (Fig. 1g–i). The vertical wax crystallites had different sizes and formed orderly rows. In turn, the *V. lantana* cuticle exhibited parallel striae and only horizontally oriented wax platelets (Fig. 1j, k). The epidermis of both species had stomata, which were nearly round and located on small protuberances in *V. opulus* (Fig. 1i–m) and slightly elongated and located at the epidermis level in *V. lantana* (Fig. 1n, o). The stomata in both species were either open or filled with epicuticular wax.

**Fig. 2:**
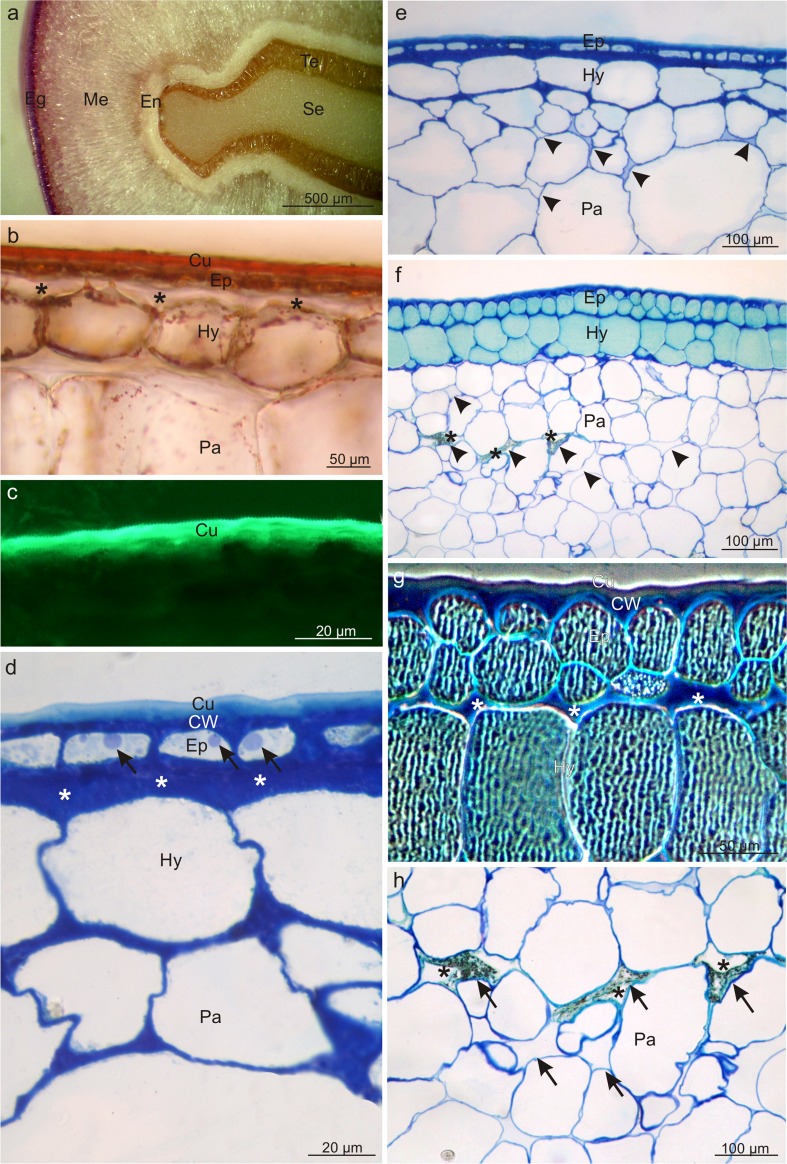
Cross sections through the pericarp of *V. opulus* (**a**–**e**) and *V. lantana* (**f**–**h**) fruits. **a** Visible fragment of the pericarp and drupe seed. **b** Section stained with Sudan III; note the intensely stained cuticle. **c** Autofluorescence of cuticle. **d** Note the thickened cuticle and the periclinal walls of the hypodermis and epidermis *(asterisks)*, and the lipid droplets *(arrows)* in the epidermis. **e** Visible cells of mesocarp parenchyma with small intercellular spaces *(arrowheads)*. **f** Note the intensely stained epidermis and hypodermis cells as well as intercellular spaces *(arrowheads)* in the mesocarp sometimes filled with a dark insoluble material *(asterisks)*. **g** In the exocarp cells, visible tannin and/or anthocyanin complexes forming a characteristic pattern (polarised light). Note the thickened periclinal cell walls of the epidermis and hypodermis *(asterisks)*. **h** Cells of mesocarp parenchyma with large intercellular spaces *(arrows)* sometimes filled with a dark, insoluble material *(asterisks)*; *Eg* exocarp, *Me* mesocarp, *En* endocarp, *Te* testa, *Se* seed, *Cu* cuticle, *CW* cell wall, *Ep* epidermis, *Hy* hypodermis, *Pa* parenchyma

The pericarp in the drupes of both *Viburnum* species consisted of a skin (exocarp) formed of the epidermis and hypodermis, a fleshy mesocarp, and a lignified endocarp (Fig. 2a, e). The epidermal cells were five- or six-sided polygons in top view in both species. The cross-section revealed epidermis with a thickened outer wall covered by a massive cuticle layer, which stained when treated with Sudan III and IV and emitted intense autofluorescence (Fig. 2b, c). The cuticle thickness in *V. opulus* was by 18% lower than that of the layer in *V. lantana*, whereas the thickness of the outer epidermis wall was by 48% higher in *V. opulus*. Moreover, the epidermis cells in *V. opulus* were shorter and narrower in the cross sections than those in *V. lantana* (Table 1).

The single-layered or sometimes double-layered (in *V. lantana*) hypodermis was composed of collenchyma cells with thickened periclinal walls adjacent to the epidermis, particularly in *V. opulus* (Fig. 2b, d–g). The walls were almost threefold thicker in *V. opulus* than in *V. lantana*. Additionally, the hypodermis cells in *V. opulus* had a greater width and height than in *V. lantana* (Table 1, Fig. 2d–g). The epidermis and hypodermis cells in *V. lantana* exhibited tannin and/or anthocyanin complexes filling the vacuoles and forming a characteristic openwork pattern (Fig. 2f, g).

Due to the asymmetrical location of the drupelet to the centre of fruit symmetry, the number of mesocarp layers in both *Viburnum* species varied considerably along the drupe periphery, but did not differ between the analysed taxa. Parenchyma cells in the cross section were characterised by a nearly one third greater height in *V. opulus* than in *V. lantana* (Table 1, Fig. 2e–h). Intercellular spaces in *V. lantana*, which were visible between the parenchyma cells were relatively numerous, had greater sizes and irregular shapes, and were filled with a dark insoluble material (Fig. 2 f, h). The fruit parenchyma also exhibited smaller and larger vascular bundles in both species, and calcium oxalate crystals were detected in deeper parenchyma layers in *V. lantana*.

### Fruit pigments and location of other metabolites

**Fig. 3:**
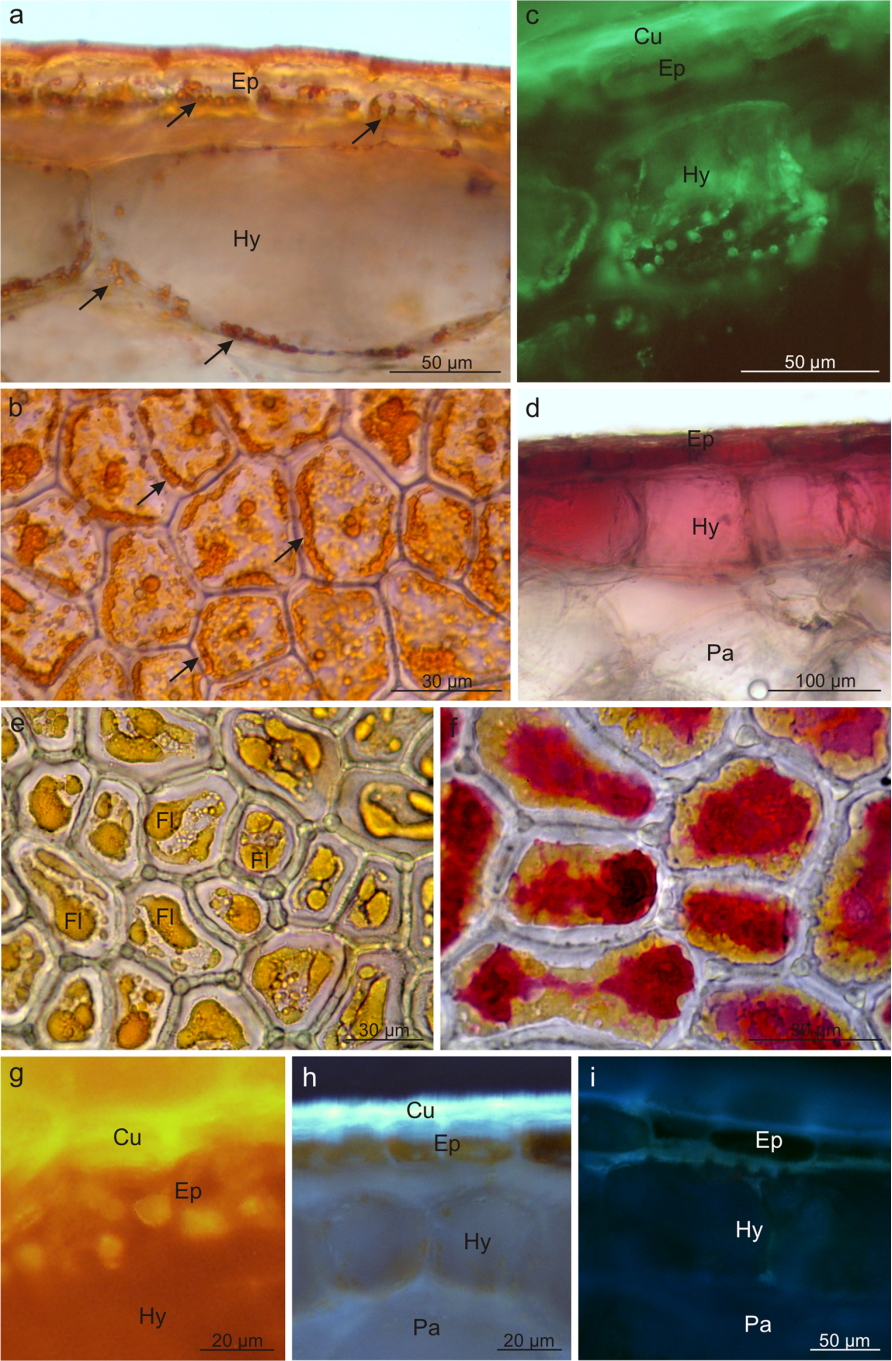
Location of pigments in the drupe skins of *V. opulus* (**a**–**c**, **e**–**h**) and *V. lantana* (**d**, **i**). **a**, **b** Chromoplasts *(arrows)* with carotenoids in skin cells; **a** cross section, **b** top view. **c** Visible carotenoid autofluorescence in the hypoderm. **d** Anthocyanins in skin cells. **e** Flavonoids in the epidermis (*top view*). **f** Flavonoids and anthocyanins in the epidermis (*top view*). **g**–**i** Flavonoids under fluorescence in the cuticle, cell walls, and epidermis cells; *Cu* cuticle, *Ep* epidermis, *Hy* hypodermis, *Pa* parenchyma, *Fl* flavonoids

Three types of pigments were detected in the fruits of the analysed *Viburnum* species: carotenoids, flavonoids, and anthocyanins. They were mainly located in the fruit skin and, less frequently, in the mesocarp cells. The *V. opulus* fruits had a coral colour due to the presence of flavonoids and carotenoids; in turn, anthocyanins, which masked the presence of carotenoids, dominated in *V. lantana*. Carotenoids filled numerous chromoplasts and emitted intense autofluorescence (Fig. 3a–c). The content of carotenoids in the *V. opulus* fruits was 10-fold higher than that in *V. lantana* (Table 2). Anthocyanins were dissolved in the vacuoles of the exocarp and peripheral mesocarp cells in *V. lantana* (Fig. 3d); in *V. opulus*, in turn, the pigments were usually located in the epidermis cells (Fig. 3f). Flavonoids were detected in the vacuoles of the *V. opulus* epidermis cells (Fig. 3e–g) and in the cuticle as well as in cell walls of both species, as shown by fluorochromes, which induced a greenish-yellow secondary fluorescence of this layer (Fig. 3h, i). The quantitative analysis revealed significantly higher content of flavonoids in the *V. opulus* fruits, in comparison with *V. lantana* (Table 2).

**Table 2:** The content of some secondary metabolites in the mature fruits of *Viburnum*

Compounds (in mg·100 g^−1^)	*V. opulus*	*V. lantana*
Carotenoids	1.50 ± 0.41	0.15 ± 0.06
Polyphenols	12.30 ± 0.35	5.16 ± 0.15
Flavonoids	0.18 ± 0.04	< 0.023

**Fig. 4:**
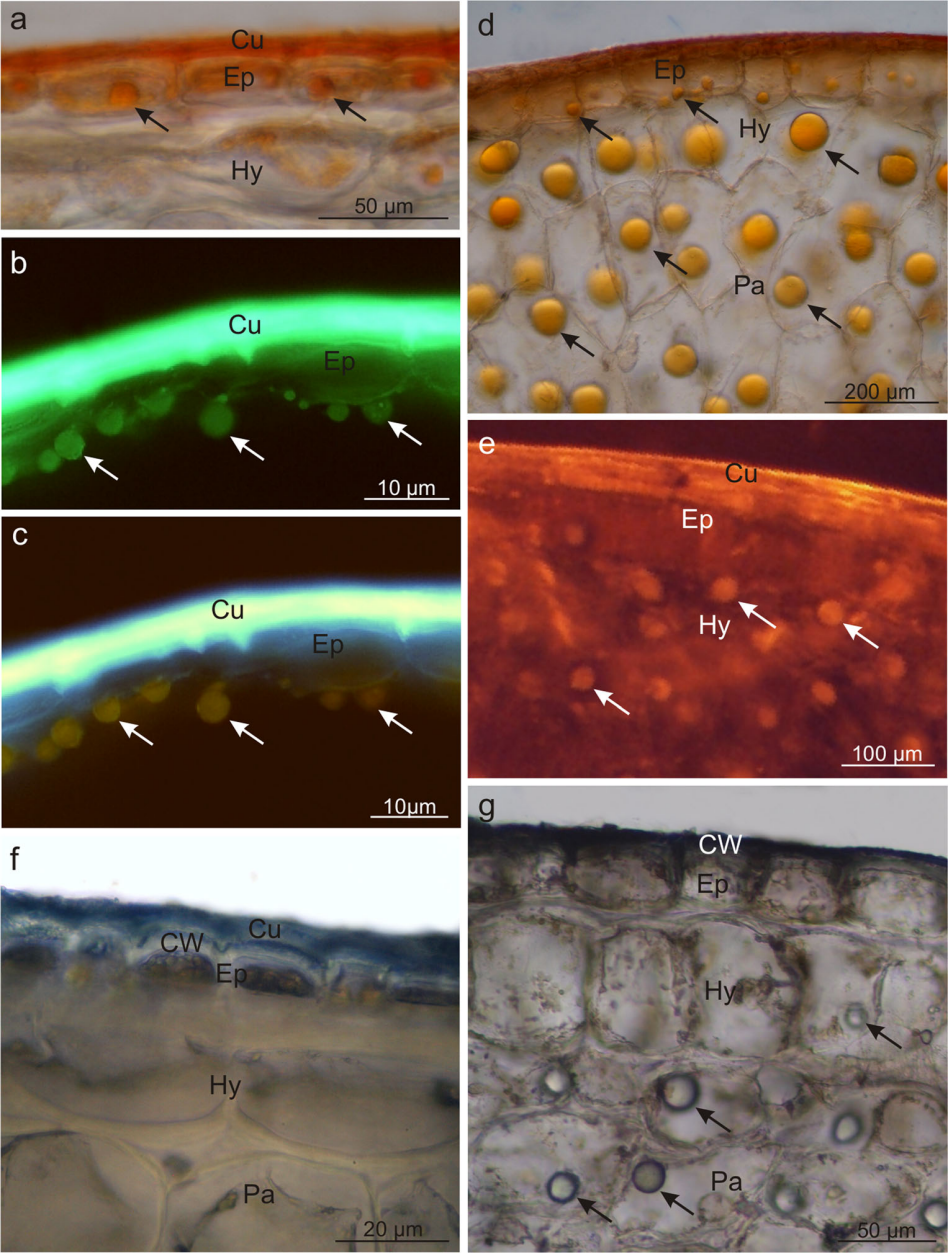
Fresh cross-sections across *V. opulus* (**a**–**c**, **f**) and *V. lantana* (**d**, **e**, **g**) pericarp subjected to different histochemical tests. **a**, **d** Staining with Sudan III; visible stained cuticle and lipid droplets *(arrows)*. **b** Staining with Neutral Red under fluorescence; lipids visible in the cuticle and in the form of droplets *(arrows)*. **c** Staining with antimony trichloride under fluorescence; steroids present in the lipid droplets *(arrows)* and epidermis. **e** Autofluorescence of lipid droplets *(arrows)*. **f**, **g** Staining with Nadi reagent; essential oils presence in the cell wall and cuticle and in lipid droplets *(arrows)*; *Cu* cuticle, *Ep* epidermis, *Hy* hypodermis, *Pa* parenchyma, *Td* tannin deposit, *Ld* lipid drop

Fluorescent microscopy and histochemical tests demonstrated that the skin cells in both species contained lipid droplets; they were less numerous and had smaller sizes in *V. opulus* and abundant and large in *V. lantana* (Fig. 4). The lipid droplets were present only in the epidermis cells in *V. opulus* (Fig. 4a–c) and in all pericarp layers in *V. lantana* (Fig. 4d–f). The lipids exhibited staining with Sudan III, IV, Nile Blue, and autofluorescence and fluorescence in the presence of Neutral Red (Table 3, Fig. 4a–e). Moreover, the cuticle and the outer wall of the epidermis cell in both species as well as the lipid droplets in *V. lantana* contained essential oils, which was confirmed by the staining reaction with Nadi reagent (Fig. 4f, g). In turn, the fluorescence of the lipid droplets in the presence of antimony trichloride in *V. opulus* indicated the presence of steroids (Table 3, Fig. 4c). The foam test confirmed the presence of saponins in the mesocarp in the drupes of both taxa but did not show the presence of these glycosides in the fruit skin. Based on the foam height, the content of saponins in *V. lantana* was found to be slightly higher than that in *V. opulus* (Table 3).

**Table 3:** Metabolites identified in *Viburnum* fruits by histochemical, fluorescence, and foam tests and their location and content

Metabolite	Reagent	*V. opulus*	*V. lantana*
Total lipids	Sudan III; Sudan IV; Neutral Red under fluorescence	+s	++sm
Acid and neutral lipids	Nile Blue	+s	++sm
Essential oils	Nadi reagent	++s	++sm
Steroids	Antimony trichloride under fluorescence	++s	±s
Carotenoids	Autofluorescence	++sm	+s
Saponins	Foam test	+m	+m
Polyphenols	Ferric trichloride	++s	+sm
Tannins	Potassium dichromate	+s	+s
Flavonoids	Aluminium chloride and magnesium acetate under fluorescence	++s	±s
Starch	IKI solution	−	−
Pectins and mucilage	Ruthenium Red	++sm	+sm
Proteins	IKI solution	−	+sm

(−) absent, (±) scare, (+) present, (++) very intense present, *s* skin, *m* mesocarp

**Fig. 5:**
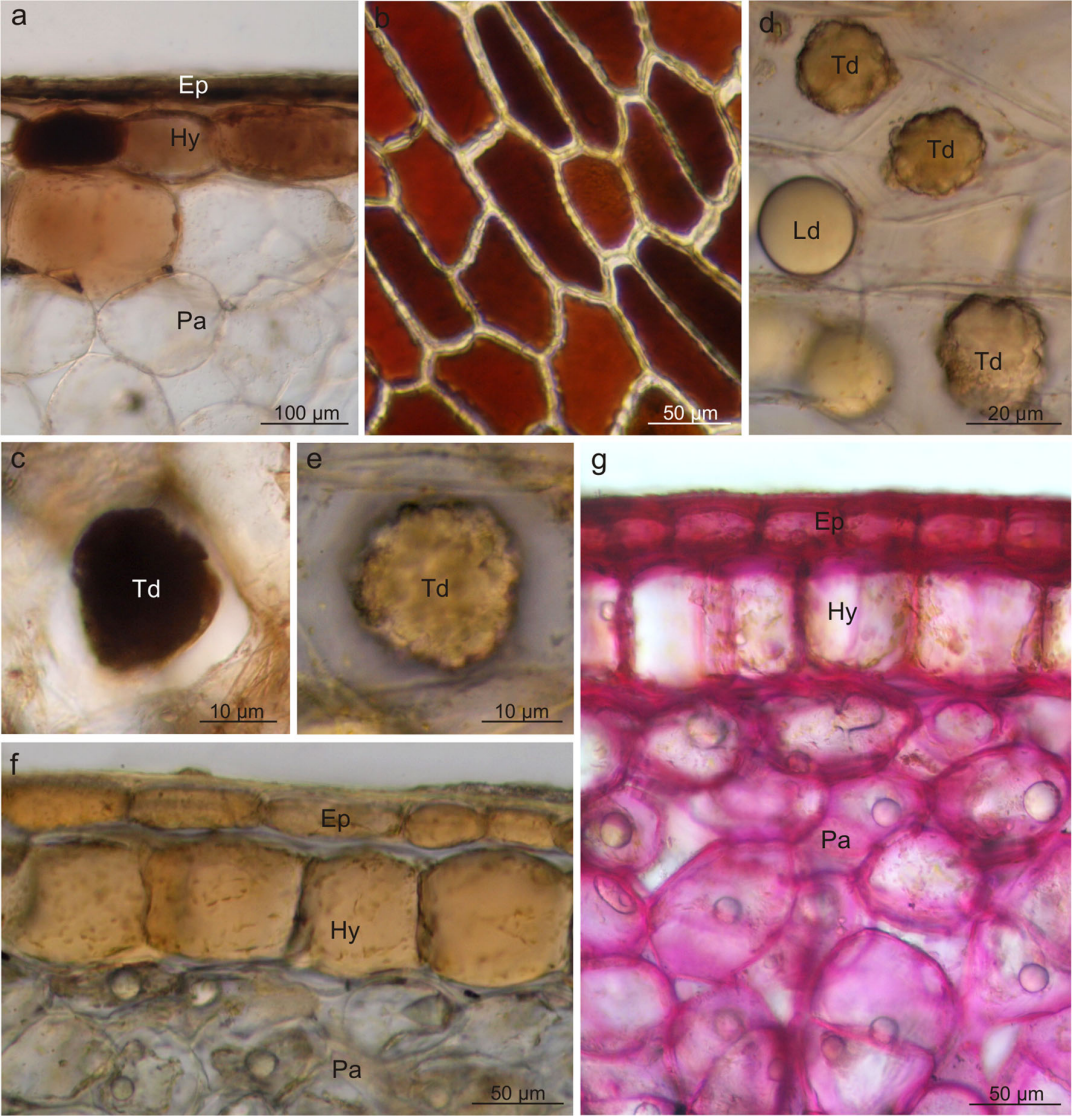
Fresh cross-sections across *V. opulus* (**a**) and *V. lantana* (**b**–**g**) fruits subjected to different histochemical tests. **a**, **c** Staining with potassium dichromate; tannins present in epidermis and hypodermis cells and in condensed deposits. **b** Staining with ferric chloride; polyphenols visible in epidermis cells. **d**–**f** Staining with the IKI solution; proteins accumulated in epidermis and hypodermis cells and in condensed deposits. **g** Staining with Rhutenium Red; pectins present in cell walls; *Ep* epidermis, *Hy* hypodermis, *Pa* parenchyma, *Td* tannin deposit, *Ld* lipid drop

The histochemical reactions showed that polyphenols, in particular tannins, were located mainly in the fruit skin but rarely in the mesocarp. After application of potassium dichromate and ferric chloride, the polyphenols were stained with intense brown colour (Fig. 5a–c). Tannins were dissolved in the vacuolar sap; however, in *V. lantana*, tannins were also visible as sediments and/or large condensed deposits (Fig. 5c–e). The *V. opulus* fruits exhibited twofold higher content of polyphenols than the *V. lantana* drupes (Table 2).

Moreover, the epidermis, hypodermis, and some parenchymal cells located under the skin as well as polyphenol deposits in *V. lantana* were stained straw-yellow colour when treated with IKI, which revealed the presence of proteins in the deposits and pericarp tissues mentioned above (Table 2, Fig. 5d–f). The fruits of the analysed *Viburnum* fruits, especially *V. opulus*, are also a rich source of pectins; this was confirmed by the reaction with Ruthenium red, which stained intensely the walls of all living cells of the drupe pericarp (Fig. 5g).

### Ultrastructure of skin cells (TEM)

**Fig. 6:**
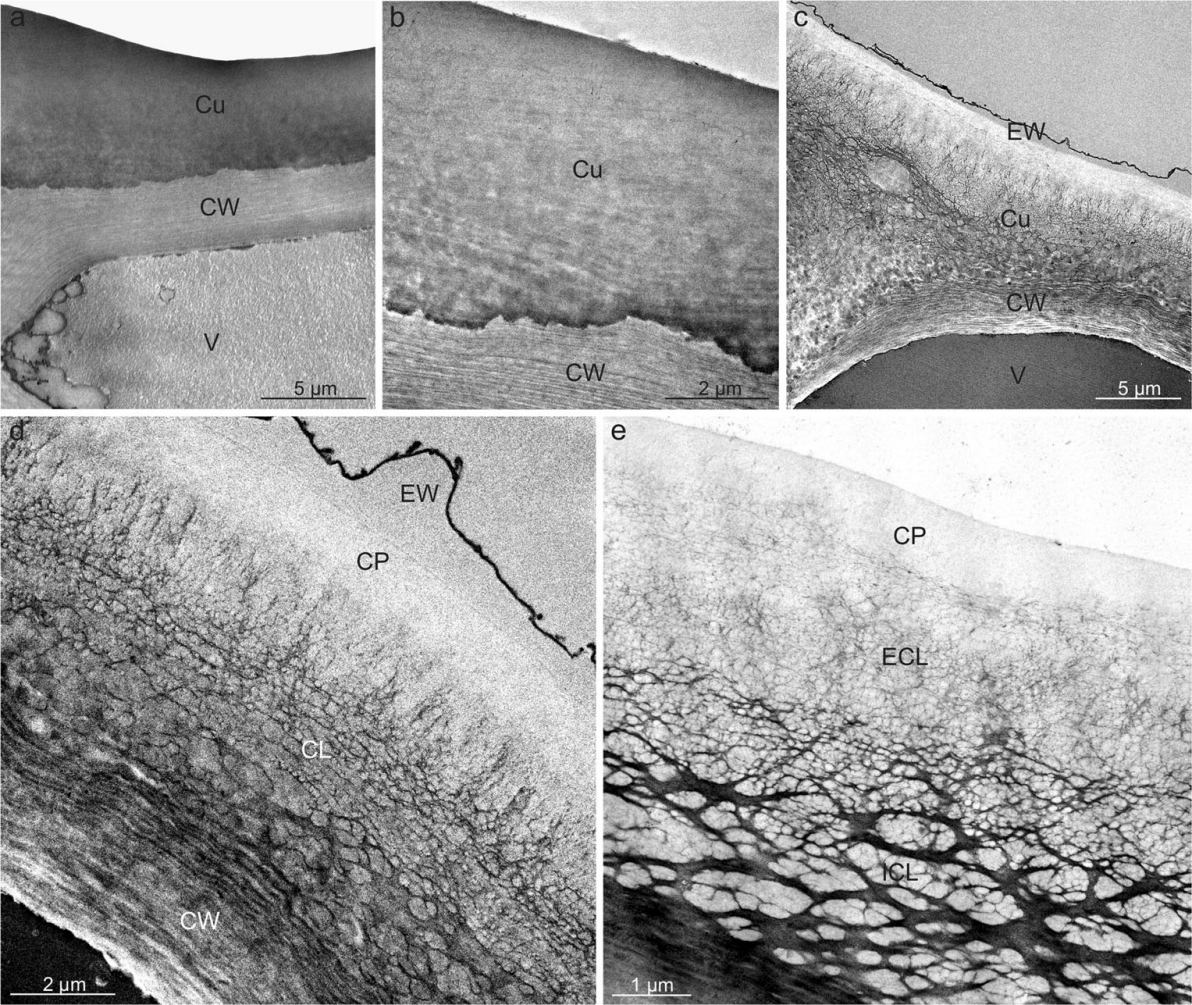
Cuticle on the *V opulus* (**a**, **b**) and *V. lantana* (**c**–**e**) fruit surface. **a** Fragment of epidermis cell with the cuticle. **b** Amorphous cuticle on the fruit surface. **c** Fragment of epidermis cell with a cell wall, cuticle, and epicuticular waxes. **d**, **e** Visible cuticle with amorphous cuticle proper and a reticulate cuticular layer with numerous polysaccharide fibres. Note the layer of epicuticular waxes; *EW* epicuticular wax, *Cu* cuticle, *CW* cell wall, *CP* cuticle proper, *CL* cuticular layer, *ECL* external cuticular layer, *ICL* internal cuticular layer, *V* vacuole

The cuticle on the surface of ripe *V. opulus* fruits was characterised by an amorphous structure throughout its thickness (Fig. 6a, b), whereas its structure varied distinctly in *V. lantana* (Fig. 6c–e). The ca. 500-nm thick outer layer, the so-called cuticle proper, in this species had a reticulate structure. Within the cuticle layer, located under the cuticle proper, a reticulate cuticle with small, densely arranged polysaccharide fibres formed an external layer, whereas the layer adjacent to the epidermis cell wall, the so-called internal layer, was characterised by the presence of large, massive fibres forming an orderly, usually periclinally oriented network. The anticlinal fibres were substantially thinner (Fig. 6c–e). A 0.4-to 0.6–μm-thick layer of epicuticular waxes was present on the surface of the *V. lantana* cuticle (Fig. 6c, d).

**Fig. 7:**
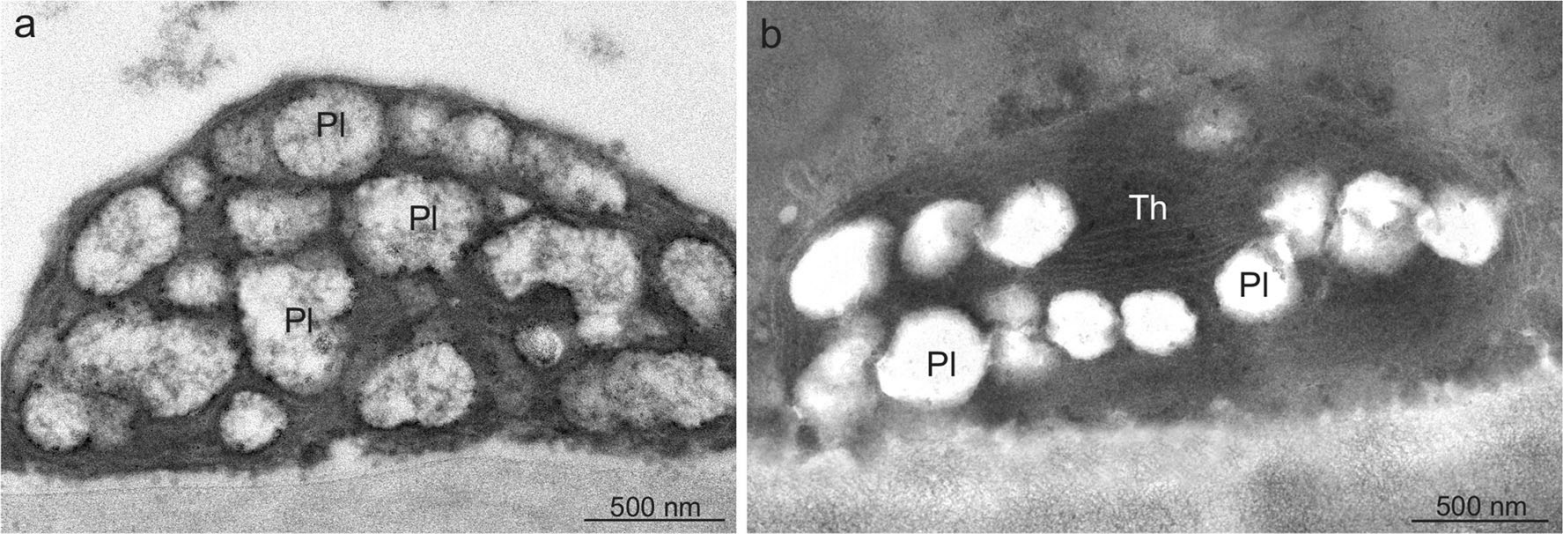
Chromoplasts in the drupe epidermis cells of *V. opulus* (**a**) and *V. lantana* (**b**). **a** Visible oval chromoplast with numerous plastoglobules. **b** Lenticular chromoplast with plastoglobules and thylakoid remnants; *Pl* plastoglobule, *Th* thylakoids

The epidermis and hypodermis cells in both species exhibited small, oval, or lenticular chromoplasts, which especially in *V. opulus* were characterised by loss of organisation and internal membrane structure and the presence of numerous, often merging vesicles (plastoglobules) with irregular shapes and usually electron-transparent content or an empty interior (Fig. 7a). In turn, the *V. lantana* chromoplasts were often elongated and, more frequently than in *V. opulus*, exhibited some small aggregated groups of thylakoid residues (Fig. 7b).

**Fig. 8:**
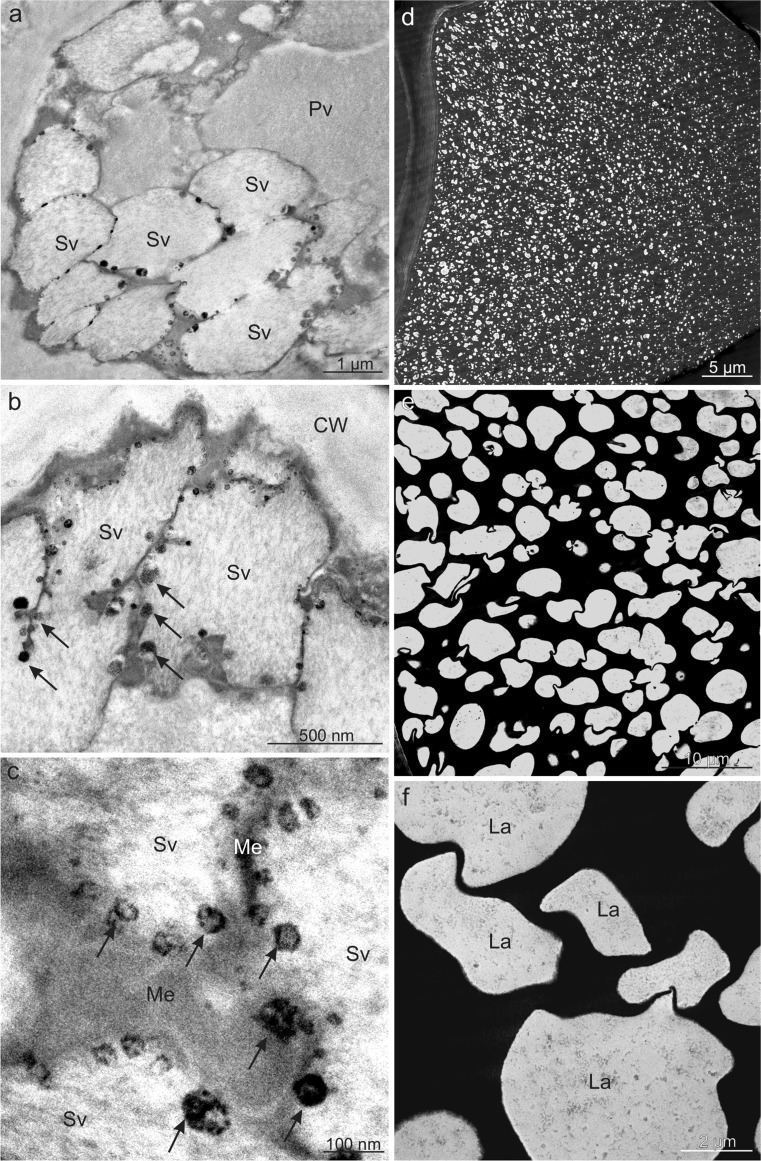
Ultrastructure of fruit epidermis cells of *V. opulus* (**a**–**c**) and *V. lantana* (**d**–**f**) in TEM. **a**, **b** Visible numerous secondary vacuoles near the cell wall with electron-dense tannin deposits (*arrows*) near membranes. **c** Tannin deposits (*arrows*) located around membranes of secondary vacuoles. **d**, **e** In the vacuole, electron-dense tannin complexes with numerous lacunae. **f** Vacuolar lacunae filled with flocculent content; *CW* cell wall, *Pv* primary vacuole, *Sv* secondary vacuole, *Me* membrane, *La* lacunae

An internal membrane system forming characteristic compartments (secondary vacuoles) was observed in the primary vacuoles of epidermis cells in *V. opulus* (Fig. 8a–c). Numerous small (100 nm and less) electron-dense tannin deposits were located near the membranes of both types of vacuoles (Fig. 8a–c). In turn, the vacuoles in *V. lantana* hypodermis and/or epidermis cells exhibited electron-dense content, which had a phenolic nature, contained numerous electron-transparent vesicles (lacunae) with varied sizes and shapes filled with flocculent content (Fig. 8d–f).

## Discussion

### Fruit morphology

*V.opulus* and *V. lantana* differed in the number of fruits in the inflorescence as well as the ripening period, colour, and shape of the drupes. The *V. opulus* shrubs were characterised by a lower number of fruits per raceme as well as larger and heavier drupes than in *V. lantana*. Some researchers report similar results concerning the number and size of fruits in the analysed *Viburnum* species to those obtained in the present study (Snow and Snow 1988; Akbulut et al. 2008). Other authors claim that the number of fruits produced in the inflorescence or on the shoot in *Viburnum* largely depends on insolation, whereas fruit weight and size in this species are modified by environmental and climate conditions as well (Kollmann and Grubb 2002, Česonienė et al. 2008). A similar diversity was found for the drupelets of the *Viburnum* species examined in this study, which were characterised by a different colour, shape, and surface as well as different proportions of the drupelet in the total fruit weight. Moreover, the *V. opulus* and *V. lantana* endocarps had different hardness although their thickness was similar. The *V. lantana* endocarp was formed of a larger number of layers of sclereids with thicker cell walls, which increased the content of lignin in this layer and, hence, the hardness of the drupelet in this species. The differences in the morphology of *V. opulus* and *V. lantana* drupelets have also been reported by other authors (Cornelissen et al. 1996, Kollmann et al. 1998). A large diversity of the structure and thickness of the drupe endocarp and seed testa in various *Viburnum* species and *Diervilla* and *Lonicera* clads, which are members of a closely related sister family Caprifoliaceae, has been described by Jacobs et al. (2008, 2009), who claim that the endocarp and seed testa structure are important taxonomic features that can be helpful in explanation of the evolution and phylogeny of these taxa.

### Skin microstructure

The fruit surface in the analysed *Viburnum* species exhibited a considerable diversity of the crystalline wax structure and the cuticle structure and thickness. Crystalline wax in the form of horizontally and vertically oriented platelets was observed on the surface of the *V. opulus* drupes, whereas *V. lantana* exhibited only horizontal crystallites. In turn, the considerably thinner cuticle on the *V. opulus* drupes was characterised by an amorphous structure, and the thick cuticle layer in *V. lantana* had a reticulate structure and contained numerous polysaccharide fibres. The authors of the present study observed rapid wilting and softening of ripe *V. lantana* fruits, which made the surface wrinkly, while the *V. opulus* drupes retained firmness and attractiveness for a long time. Various authors report that the crystalline wax formed exclusively by horizontal platelets was less efficient in limiting fruit transpiration than vertical platelets, similar to the polysaccharide fibres in the cuticle, which promote a faster rate of water loss and fruit wilting and shrinkage than an amorphous cuticle deprived of this type of fibres (Peschel et al. 2003, Jeffree 2006). The drupes of *Sambucus* are characterised by absence of a wax coating as well, whereas an intense wax layer is visible on the surface of *Lonicera* berries (Hummer et al. 2012)*.* Knoche (2015) reported that the presence of epicuticular waxes on the fruit surface is crucial for limitation of water loss, while transpiration through a cuticle devoid of wax can be highly intense. Moreover, Riederer and Schreiber (1995) and Konarska (2013) have shown that the thickness of the cuticle on the surface of other fruits is not correlated with cuticular water permeability. The exocarp of the fruits in the analysed *Viburnum* species was composed of one epidermis layer and usually single-layered hypodermis, whose walls in *V. lantana* were slightly thickened in comparison to *V. opulus*. The thinner hypodermis walls in *V. lantana* drupes may be the cause of rapid shrinking of the fruit surface in this species. Various authors claim that textural features of fruit hypodermis, e.g. wall thickness, cell-to-cell contact, and the amount of air spaces, modify strongly fruit resistance to compression and deformation (Allan-Wojtas et al. 2001; Taiz and Zeiger 2002; Chiabrando et al. 2009). Similar cell layers in the exocarp of *Sambucus nigra* (Adoxaceae) drupes were observed by Vandishev et al. (2013), who distinguished one–two layers of oval thin-walled cells with no intercellular spaces between them.

### Lipophilic components

Various types of lipophilic components, i.e. carotenoids, essential oils, steroids, and saponins, were accumulated in the drupes of *V. opulus* and *V. lantana*. Similar to other fruit pigments, carotenoids were located mainly in the exocarp cells. The enhanced carotenoid accumulation in the ripening *Viburnum* drupes proceeded in parallel with transformation of chloroplasts into chromoplasts. The chromoplasts had a form of globoid structures and exhibited partial or complete disappearance of thylakoid membranes. The plastids contained numerous plastoglobules, sometimes with an empty interior, which may have been caused by removal of carotene during fixation. As suggested by various authors, the chloroplasts-chromoplast transformation is the most frequent modification of plastids taking place in ripening fruits of various species (Schweiggert et al. 2011; Vázquez-Gutiérrez et al. 2011). Researchers who studied the interconversion of chloroplasts into chromoplasts noted that the process was manifested by degradation of the photosynthetic machinery and membrane degradation (Bonora et al. 2000; Fu et al. 2012). Blebs, numerous plastoglobules, and minute osmophylic globules were visible inside the degraded chloroplasts. Bréhélin and Kessler (2008) and Egea et al. (2010) suggest that the numerous enlarged plastoglobulins in forming chromoplasts participate in carotenoid sequestration. A similar chromoplast structure as that observed in *Viburnum* was reported by other authors studying different fruits (Masia et al. 1992; Camara et al. 1995; Vázquez-Gutiérrez et al. 2011). As suggested by Ytterberg et al. (2006) and Walter and Strack (2011), carotenoid-accumulating structures can have a globular, crystalline, membranous, fibrillar, or tubular form in different plants. The authors of the present study have shown that the carotenoid content in the *V. opulus* fruits was 1.5 mg 100 g^−1^, which was 10-fold higher than that in *V. lantana*. Different authors report a wide range of the carotenoid content from 1.25 to 6 mg 100 g^−1^ in *V. opulus* and a substantially lower content in *V. lantana* (Schaefer et al. 2008; Česonienė et al. 2010). Neuhaus and Emes (2000) and Barsan et al. (2010) have shown that chromoplasts are not only sites of biosynthesis of carotenoids but also organelles involved in the synthesis of sugars, lipids, aromatic compounds, vitamins, and hormones. They also protect plants from high light stress and contribute to seed dispersal by providing the fruits with an attractive colour (Howitt and Pogson 2006).

Essential oils and steroids were accumulated in the form of lipid droplets in the cuticle and epidermal cell walls in both species and formed lipid droplets in all pericarp layers in *V. lantana*. In turn, saponins were detected only in the fruit mesocarp in both species. Different authors confirm the presence of these metabolites in *Viburnum* fruits but do not show their location site (Yilmaz et al. 2008; Kraujalytė et al. 2012). In contrast, Babuśka-Roczniak and Roczniak (2010) have proved that phytosterols in other plants can be part of cell membranes and are present in the cytoplasm or lipid vacuoles in a free form and bound with glycosides, saponins, terpenes, and reserve lipids. Other authors have shown high content of lipids in *Viburnum* seeds as well as in *Sambucus* and *Lonicera* seeds (Palíková et al. 2008; Özrenk et al. 2011; Dulf et al. 2013)*.*

### Polyphenols

The presence of several groups of polyphenols, i.e. flavonoids, anthocyanins, and tannins, was detected in the drupes of the analysed *Viburnum* species. The polyphenols were mainly located in the fruit skin. The *V. opulus* drupes exhibited a twofold higher polyphenol content than in *V. lantana*. The presence of polyphenols in the epicuticular waxes and cuticle as well as the skin of various fruits has been revealed by other authors as well (Usenik et al. 2013; Konarska 2014a, 2015). A similar amount of polyphenols in *V. opulus* fruits was shown by Rop et al. (2010) and Moldovan et al. (2012), whereas Sagdic et al. (2006) and Özrenk et al. (2011) demonstrated several-fold higher values of total phenolic compounds. In turn, Česonienė et al. (2010) reported that the content of phenolic compounds in fruit *V. opulus* cultivars had different values in the range from 75.3 to 146.0 mg 100 g^−1^. Kollmann and Grubb (2002) suggested that the variable content of different metabolites in *Viburnum* fruits depends on environmental and climate conditions.

The authors of the present study have shown that flavonoids were present in the epidermis cells in the *V. opulus* drupes and in the cuticle in both species, and their total content in *V. opulus* was several-fold higher than in *V. lantana*, i.e. 0.18 mg 100 g^−1^ *.* The results of studies conducted by other authors confirm the presence of flavonoid pigments, i.e. quercetin and flavones, in *V. opulus* and *V. lantana* fruits (Velioglu et al. 2006) and as well as catechins, i.e. flavonoids with antioxidant properties (Yunusova et al. 2004). In turn, Rop et al. (2010) and Erdogan-Orhan et al. (2011) demonstrated that the content of flavonoids in *V. opulus* fruits was 0.1–0.5 mg 100 g^−1^, whereas *V. lantana* fruits contained substantially lower amounts of these metabolites (0.05–0.2 mg 100 g^−1^). As suggested by various researchers, the role of flavonoids contained in fruits is to provide protection of the fruit photosynthetic apparatus against UV-B-induced damage at strong sunlight (Solovchenko and Schmitz-Eiberger 2003) and to increase the cuticle stiffness (Tsubaki et al. 2012).

Based on the results of histochemical tests and LM and TEM observations, it was shown that the skin cell vacuoles and intercellular spaces in the mesocarp of the examined *Viburnum* species serve as tannin accumulation sites. Besides their soluble form, insoluble sediments of these compounds as well as condensed, electron-dense, different-size accretions, the so-called proathocyanins, were observed. A similar location and form of tannins in the fruit pericarp of various species have also been observed by other authors (Hammouda et al. 2014; Konarska 2013, 2014b). Some researchers suggest that tannins can form stable complexes with proteins, carbohydrates, and fats (Jakobek 2015) as well as heavy metals, mucilage, and pectins (Ramaswamy et al. 2013; Zhao et al. 2013). Literature data indicate that tannins are particularly abundant in young, unripen fruits and are responsible for their astringent taste; during the fruit ripening period, their content declines as the compounds are hydrolysed into various sugars and acids or converted into other forms of secondary metabolic compounds (Robil and Tolentino 2015, Tessmer et al. 2016). Many authors agree that tannins repel herbivores and protect plants from pathogens (Lattanzio et al. 2006), and their presence can determine fruit attractiveness to potential seed dispersers (Kollmann and Grubb 2002). However, Cazetta et al. (2008) have detected high tannin contents in *Viburnum* drupes also in ripen fruits as the major cause of the unattractiveness of *Viburnum* fruits to frugivores (birds and mammals); therefore, the fruits can persist on the shrubs until spring.

In the skin cells of the analysed *Viburnum* drupes, anthocyanins were dissolved in the cell sap (*V. opulus*) and/or formed insoluble complexes with other metabolites, e.g. tannins and/or proteins (*V. lantana*)*.* Similar location and forms of anthocyanins were described by Bernal et al. (2015) and Franceschinis et al. (2015), whereas the formation of concentrated spherule structures, containing anthocyanins at high concentrations, the so-called anthocyanic vacuolar inclusions, was reported by Conn et al. (2010) and Mizuno et al. (2015) in analyses of the skin of other fruits. Different authors show that anthocyanins are synthesised by multi-enzyme complexes that are localised at the cytoplasmic face of the endoplasmic reticulum (Winkel 2004); next, they accumulate at higher concentrations in several vesicles of different sizes in the cytoplasm and then they actively move alongside the tonoplast and accumulated in the vacuole (Gomez et al. 2011). Similar to *Viburnum* fruits, the fruit of various *Sambucus* and *Lonicera* species are a rich source of anthocyanins and phenolic compounds and exhibit a strong antioxidant capacity (Chaovanalakit et al. 2004; Wu et al. 2004; Ozgen et al. 2010).

## Conclusions

The investigations have revealed a number of differences in the micromorphology, anatomy, and ultrastructure of *V. opulus* and *V. lantana* fruits. The differences involved fruit cuticle thickness and structure, cuticular wax structure, thickness of pericarp layers and cells contained therein, and thickness of the cell walls in these tissues. There were also differences in the content of pigments responsible for the fruit colour as well as the occurrence, location, and content of biologically active compounds. It has been documented that a majority of the metabolites analysed were located in the fruit skin. The *V. opulus* fruits have been shown to exhibit greater firmness and durability as well as health-enhancing values due to their content of carotenoids, flavonoids, and polyphenols, whereas the *V. lantana* drupes contained greater amounts of proteins, essentials oils, and saponins. Carotenoids, anthocyanins, flavonoids, and tannins contained in the fruits of the analysed *Viburnum* species are valuable human and animal health-promoting compounds, as they have potent antioxidant activity that could reduce the risk of cancer and cardiovascular diseases. The results obtained represent advances in the knowledge of the *Viburnum* fruit microstructure and location of bioactive substances in two *Viburnum* species, which can provide a foundation for future studies aimed at a wider use of these metabolites for food, nutraceutical, and medicinal purposes. Furthermore, investigation of the fruit microstructure can improve our understanding of the relationship between the structure and tissue function and between the structure and accumulation of active substances.
